# Rare Presentation of Stercoral Ulcer Spontaneous Perforation Without Preceding Radiographic Evidence of Colonic Inflammation and Fecal Impaction

**DOI:** 10.7759/cureus.35678

**Published:** 2023-03-02

**Authors:** Harshavardhan Sanekommu, Andrea Morris, Sobaan Taj, Steven Imburgio, Anmol S Johal, ZakaUl Haq, Arif B Saleh, Pranav Shah, Swapnil V Patel

**Affiliations:** 1 Internal Medicine, Jersey Shore University Medical Center, Neptune City, USA; 2 Medicine, St. George's University School of Medicine, West Indies, GRD; 3 Internal Medicine, Raritan Bay Medical Center, Perth Amboy, USA; 4 Radiology, Jersey Shore University Medical Center, Neptune City, USA

**Keywords:** colitis, fecal impaction, constipation, radiographical findings, colon, stercoral ulcer perforation

## Abstract

Stercoral ulcers are caused by persistent fecal impaction. A life-threatening consequence of stercoral ulcers is colonic perforation, which is rare. A high index of clinical suspicion should be held for patients with stercoral ulcer, as colonic perforation is a medical emergency, requiring immediate surgical intervention. Here, we report a case of a 45-year-old female admitted with sepsis of unknown picture and subsequently developed stercoral ulcer perforation (SUP), diagnosed intraoperatively, without prior radiographic evidence of colonic inflammation. She was successfully managed with emergency laparotomy and left colectomy with sigmoid colectomy.

## Introduction

Stercoral colitis is a rare inflammatory process most commonly of the rectosigmoid colon related to prolonged fecal impaction associated with chronic constipation and colonic dysfunction [1]. Ischemic necrosis and stercoral ulcer formation can result, with the potential for subsequent colonic perforation [2]. Stercoral ulcer perforation (SUP) has been reported in fewer than 150 cases worldwide by 2018 [2]. Perforation with contamination of the peritoneal cavity has a high morbidity and mortality rate, and rarely can be present in patients not exhibiting symptoms of peritonitis [3]. We report a case of SUP diagnosed intraoperatively in a patient with benign radiographic findings prior to the perforation.

## Case presentation

**Figure 1:**
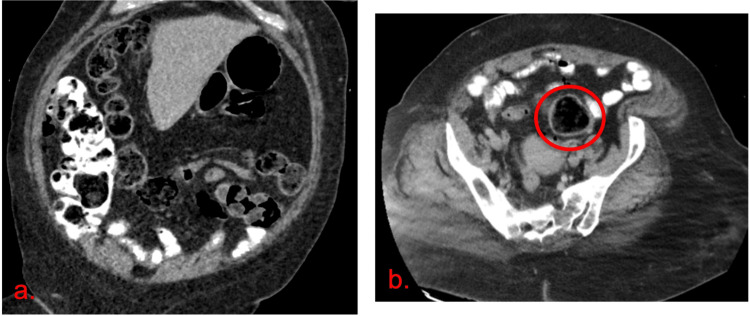
CT abdomen/pelvis. (a) Stool throughout the colon with contrast penetrating through the entire length of colon. (b) Sigmoid colon (red circle) without signs of colitis or fecal impaction.

We report a case of a 45-year-old female with past medical history of chronic constipation from methadone, gastroesophageal reflux disease presented with diffuse lower abdominal pain that began one day ago. Her last bowel movement was eight days ago. On presentation, blood pressure 112/58 mmHg, heart rate 106/min, saturating well on room air, and afebrile. On abdominal exam, tenderness prominent in the lower quadrants with guarding and diffusely dull resonance. Labs were significant for white blood cell (WBC): 16,400/µL with 90% neutrophil percent, hemoglobin 12.1 g/dL, platelets 275,000/µL, and lactic acid 2.2 mmol/L. Urinary analysis was negative for any infectious etiology. CT of the abdomen and pelvis with contrast showed large stool throughout the colon without any acute colonic pathology (Figure 1a,b).

She was started on Piperacillin/Tazobactam and was admitted for sepsis of unknown origin.

**Figure 2:**
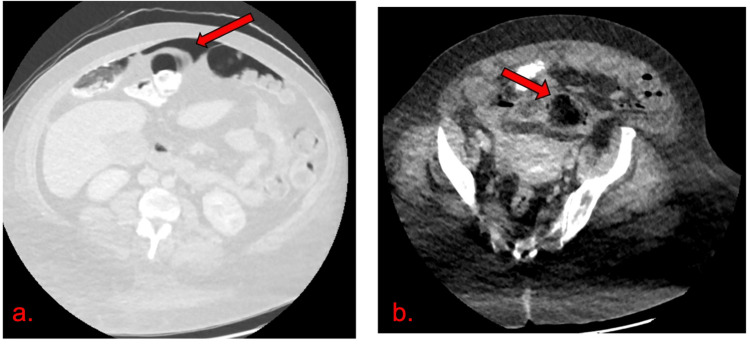
CT angiogram. (a) Evidence of pneumoperitoneum seen within the intra-abdominal cavity using lung window (red arrow). (b) Localized sigmoid inflammation, most likely site of perforation (red arrow).

A day after admission, the patient described a popping sensation in the lower right quadrant with stabbing pain that radiates to the right clavicle. Physical exam revealed an abdomen that was diffusely distended with positive rebound tenderness, guarding, and tympanic resonance. CT angiogram of abdomen and pelvis was consistent with pneumoperitoneum with localized inflammation and perforation in the sigmoid colon (Figure 2a,b).

The patient emergently underwent exploratory laparotomy and upon entry of the peritoneal cavity, feculent and purulent fluid was encountered. A large perforated stercoral ulcer in the rectosigmoid was identified with fecalomas protruding through the perforation as well as the entire length of the left colon. Despite undergoing colonic lavage, impacted stool persisted throughout. Due to an increased risk of perforation, the patient underwent an extended left colectomy with sigmoid colectomy. Intra-abdominal cultures taken during the procedure grew polymicrobial microorganisms including those that produce extended spectrum beta-lactamases (ESBL). The patient completed a course of ertapenem and was discharged.

## Discussion

Stercoral perforation has high mortality rates [2, 4]. Early diagnosis and intervention is key in the management of bowel perforation. The single greatest risk factor is chronic constipation, present in 81% of all patients [5]. The most commonly associated drugs which precipitate constipation are: opioids, tricyclic antidepressants, and anticholinergics [1]. In this case report, we report a patient, in whom we saw the chronic opioid dependence was a major risk factor that predisposed her to chronic constipation.

Patients present with benign symptoms with non-perforated stercoral ulcers: abdominal pain, vomiting, rectal discomfort, constipation, etc. Patients with perforation have bloody stools with signs of peritoneal inflammation [1]. Our patient presented with signs of peritoneal inflammation but no bloody diarrhea. One possible explanation for this could be that the perforation was identified in its early stages before bloody diarrhea could have manifested. This change in the physical exam prompted further evaluation which revealed a life-threatening perforation.

CT abdomen has an important role in identifying this life-threatening complication of stercoral ulcers. Some of the findings are: colonic dilatation >6 cm, colonic wall thickening >3 mm, pericolonic fat stranding, mucosal discontinuity, presence of free air, free fluid, and pericolonic abscess [1, 5]. Initial CT abdomen for our patient did not show any signs of bowel inflammation, abscess, or fecal impaction to suggest stercoral ulcer, which usually precedes before perforation. CTA was ordered to rule out obstruction, mesenteric ischemia, and perforation. The patient was found to have pneumoperitoneum, requiring emergent surgery. In our literature review, there have been no cases reported with SUP that did not have radiographic evidence, especially CT abdomen, of colonic inflammation or fecal impaction. This could be because many patients were present with perforation initially and have pronounced inflammation consequently on imaging.

The diagnosis is made during exploratory laparotomy and fecalomas coming through the perforated ulcer is diagnostic [1]. Similarly, our patient was diagnosed with SUP during the exploratory laparotomy. The severity of the perforation seemed to extend beyond the local perforation as stool was found protruding through the entire length of the descending colon, necessitating left colectomy with sigmoid colectomy. Of the 91 case reports reviewed with SUP, only five case patients required more than a Hartmann procedure, like in our patient who also underwent a left colectomy [1].

Without surgical intervention, SUP has 100% mortality. Even with surgical intervention, the prognosis is poor [6-8]. The intra-abdominal cultures in our patient present another complication but one that is well described in literature is related to bowel perforation [9]. Broad spectrum antibiotics are employed and have been successful, like the ertapenem that was administered to the patient [9]. We were able to successfully escalate our case based on the change of symptoms for immediate surgical intervention.

## Conclusions

We report a rare case of stercoral perforation without radiographic studies showing stercoral colitis, ulcer, or fecal impaction that increases the risk of SUP. Diagnosis of SUP was made intraoperatively in our patient. This is the first case report with a negative CT abdomen in a patient with SUP. We conclude the importance of developing a high suspicion for complications of chronic constipation, including bowel perforation. This is especially true when there is a change of clinical symptoms, particularly with signs of peritonitis. 
